# Elucidating Volatile Flavor Profiles and Metabolic Pathways in Northern Pike (*Esox lucius*) During Superchilled Storage: A Combined UPLC-Q-TOF/MS and GC-MS Approach

**DOI:** 10.3390/foods14152556

**Published:** 2025-07-22

**Authors:** Shijie Bi, Na Li, Gao Gong, Peng Gao, Jinfang Zhu, Batuer Abulikemu

**Affiliations:** 1 College of Food Science and Pharmacy, Xinjiang Agricultural University, Urumqi 830052, China; lina201127@163.com (N.L.); gaopeng9144911@sina.com (P.G.); zjf7619@126.com (J.Z.); batur6805@126.com (B.A.); 2 College of Animal Sciences, Xinjiang Agricultural University, Urumqi 830052, China; ggao1995@163.com

**Keywords:** *Esox lucius*, superchilled storage, volatile compounds, metabolomics

## Abstract

Temperature is the most critical factor in fish preservation. Superchilled storage represents a novel technology that effectively retards quality deterioration in aquatic products. This study investigated the flavor variation patterns and deterioration mechanisms in 16 northern pike (*Esox lucius*) samples during superchilled storage (−3 °C) based on analysis using gas chromatography-ion mobility spectrometry (GC-IMS) and ultra-performance liquid chromatography–quadrupole time-of-flight mass spectrometry (UPLC-Q-TOF/MS). The results indicate that GC-MS analysis identified 25 key volatile flavor compounds. These comprised seven ketones, thirteen alcohols, aldehydes including 2-methylbutanal, esters such as 2-heptyl acetate and methyl butyrate, as well as nitrogen-containing compounds, exemplified by pyrazines and indole. Non-targeted metabolomics further revealed four pivotal metabolic pathways, glycerophospholipid metabolism, purine metabolism, the pentose phosphate pathway, and arginine biosynthesis. These metabolic pathways were found to regulate flavor changes through modulation of lipid oxidation, nucleotide degradation, and amino acid metabolism. Notably, the arginine biosynthesis pathway exhibited significant correlations with the development of characteristic cold-storage off-flavors, mediated by glutamate accumulation and fumarate depletion. This investigation provided a theoretical foundation for optimizing preservation strategies in cold-water fish species at the molecular level.

## 1. Introduction

The northern pike (*Esox lucius*), a cold-water fish of significant economic value with a circumpolar distribution in the Northern Hemisphere, is increasingly recognized as a premium aquatic product in high-end markets [1]. This status stems from its desirable attributes, including tender texture, high protein content, and abundant polyunsaturated fatty acids and essential amino acids [2]. However, its elevated moisture levels and robust endogenous enzymatic activity render it highly perishable during storage. While conventional freezing effectively inhibits microbial proliferation, substantial ice crystal formation disrupts myofibrillar structure, resulting in irreversible post-thawing quality defects such as drip loss, textural softening, and flavor deterioration. These changes significantly compromise sensory quality and commercial value [3]. According to the Food and Agriculture Organization (FAO), improper preservation of aquatic products incurs global economic losses exceeding $12 billion annually, with frozen storage-related quality deterioration accounting for approximately 30% [4]. Consequently, the development of novel preservation technologies capable of extending shelf life while maintaining fresh-like quality is urgently required.

As an emerging physical preservation technique, superchilled storage demonstrates significant advantages for aquatic products [5]. Its core mechanism involves precise temperature control to induce partial water freezing, thereby inhibiting microbial metabolism and endogenous enzyme activity while minimizing mechanical damage to muscle tissue from ice crystal formation [6]. Superchilled storage demonstrates significant environmental advantages over conventional freezing methods, primarily through a 50–70% reduction in energy consumption by operating at near-cryoscopic temperatures (−1 to −4 °C) that minimize phase change penalties [7]. Previous studies have shown that compared to refrigeration and freezing, superchilling maintained mackerel fillet quality more effectively, minimizing adverse effects on protein solubility, drip loss, and tissue softening [8]. However, existing research has predominantly focused on the effects of superchilled storage on physicochemical indices (pH, total volatile base nitrogen (TVB-N), thiobarbituric acid-reactive substances), and microbial community succession [9,10]. This focus reveals a critical knowledge gap regarding the temporal dynamics of characteristic flavor compounds, the principal chemical drivers of quality deterioration, and their underlying metabolic regulatory mechanisms.

Recent advancements in omics technologies have offered novel insights into the molecular mechanisms governing food quality deterioration processes [11]. Metabolomics, through high-throughput profiling of small-molecule metabolites, enables systematic elucidation of regulatory networks within key metabolic pathways under specific storage conditions [12,13]. Studies have revealed the coupling effects of ice storage temperature on lipid oxidation pathways and umami amino acid metabolism in rainbow trout [14]. Nevertheless, the co-evolution of metabolites and flavor compounds in northern pike under superchilled storage remains largely unexplored.

To address this gap, this study combines untargeted metabolomics and volatile flavoromics to systematically explore the interaction mechanisms between metabolites and flavor substances in northern pike during superchilled storage. This study represents the multi-omics investigation into the molecular regulatory mechanisms underpinning superchilled preservation. The findings advance the scientific understanding of low-temperature storage theory for aquatic products and provide critical data support for developing intelligent preservation technologies aimed at inhibiting spoilage pathways.

## 2. Materials and Methods

### 2.1. Raw Material and Sampling

Live specimens of *Esox lucius* were procured from the same aquaculture company at Beiyuanchun Market (Urumqi, China) in October 2024, with an average weight of 0.82 ± 0.04 kg and body length of 31.06 ± 1.58 cm. A total of 16 specimens were used in this study. The fish were immediately transported to the laboratory and humanely euthanized through percussive cranial stunning in compliance with the animal ethics guidelines, followed by gutting, washing, and filleting. Uniform dorsal muscle portions (3 × 3 × 1.5 cm) were excised from each fillet. The samples were vacuum-sealed in sterile bags and stored at −3 °C for 30 d. At each of the two time points (day 0 and day 30), the tissues were flash-frozen in liquid nitrogen and preserved at −80 °C for subsequent analysis.

### 2.2. Analysis of VOCs by GC-MS

The methodology was adapted from Chen et al. (2023) with some modifications [15]. Each sample (250 ± 5 mg) was transferred to a 20 mL headspace vial, and 10 μL of 2-octanol (10 mg/L in deionized water) was introduced as an internal standard. Volatile compound analysis was performed using headspace solid-phase microextraction gas chromatography-time-of-flight mass spectrometry (HS-SPME-GC-TOF-MS; Agilent 7890GC/5977B MSD; Agilent Technologies, Santa Clara, CA, USA). HS-SPME was carried out using an automated PAL rail system with the following parameters: incubation temperature 60 °C (preheating 15 min, extraction 30 min), followed by 4 min fiber desorption. The GC system was equipped with a DB-Wax capillary column (30 m × 250 μm × 0.25 μm; Agilent Technologies). The helium carrier gas was set at a 3 mL/min inlet purge flow and 1 mL/min constant column flow. The oven temperature program was initially held at 40 °C for 4 min, then ramped to 245 °C at a rate of 5 °C/min, with a final hold for 5 min. The instrumental parameters were configured as follows: injector, 250 °C; transfer line, 250 °C; ion source, 230 °C; and quadrupole, 150 °C. Electron impact ionization was operated at 70 eV with mass spectra acquired in scan mode (*m*/*z* 20–400), incorporating a 2.37 min solvent delay.

### 2.3. Metabolomic Analysis

#### 2.3.1. Metabolites Extraction

Based on preliminary experiments monitoring physicochemical indices in *Esox lucius* during superchilled storage, samples from days 0 and 30 were selected for metabolomic analysis. A 25 mg aliquot of *Esox lucius* tissue was weighed into a pre-chilled Eppendorf tube on ice. Homogenizing beads were added, followed by 500 μL of extraction solution (methanol: acetonitrile: water, 2:2:1 *v*/*v*/*v*) containing isotope-labeled internal standards. The mixture was vortexed for 30 s and homogenized using a bead mill homogenizer (35 Hz, 4 min, JXFSTPRP-24, Shanghai Jingxin Technology Co., Ltd., Shanghai, China). The homogenate was transferred to an ice-water bath and ultrasonicated for 5 min; this cycle was repeated thrice. Subsequently, the samples were incubated at −40 °C for 1 h. After incubation, centrifugation was performed at 13,800× *g* for 15 min at 4 °C and the resulting supernatant was transferred to a fresh vial for analysis.

#### 2.3.2. UHPLC-MS Analysis

The target compounds were chromatographically separated using a Vanquish ultra-high-performance liquid chromatography (UHPLC) system (Thermo Fisher Scientific, Waltham, MA, USA) equipped with a Waters ACQUITY UPLC BEH Amide column (2.1 mm × 50 mm, 1.7 μm, Shanghai Lianqiao Biotechnology Co., Ltd., Shanghai, China). Mobile phase A consisted of an aqueous solution containing 25 mM ammonium acetate and 25 mM ammonia, whereas mobile phase B consisted of acetonitrile. The sample tray temperature was maintained at 4 °C and the injection volume was 2 μL. Data acquisition for both MS and MS/MS analyses was performed on an Orbitrap Exploris 120 mass spectrometer controlled by the Xcalibur software (v4.4; Thermo Fisher Scientific). The operational parameters were set as follows: sheath gas flow rate, 50 mL/min; auxiliary gas flow rate, 15 mL/min; capillary temperature, 320 °C; full mass spectrometry resolution 60,000, and MS/MS resolution, 15,000. Stepped normalized collision energy (SNCE) was applied at 20, 30, and 40%. The spray voltage was optimized to +3.8 kV (positive ion mode) and −3.4 kV (negative ion mode) [16]. Technical replicates were performed in triplicate (RSD < 5%). Method validation confirmed accuracy (92–107% recovery), intra-day precision (RSD 1.2–4.8%), inter-day precision (RSD < 6.5%), and sensitivity (LODs 0.01–0.2 μg/L, LOQs 0.03–0.5 μg/L). Quality control included daily system suitability tests (retention time/intensity RSD < 3%), intermittent blank injections, and NIST SRM 1950 reference material in each batch.

### 2.4. Data Processing and Statistical Analysis

Volatile compounds were analyzed using the Chroma TOF 4.3X software (LECO Corporation, St. Joseph, MI, USA) in conjunction with the NIST database. NIST library match scores > 800 and retention index deviation ranges < 20 units. Raw chromatographic peaks were extracted with precision followed by baseline filtering and calibration. Subsequent peak alignment and deconvolution analyses were performed sequentially. Peaks were identified and their areas were integrated. Compound identification was confirmed by spectrum matching. Statistical analyses were performed using the R software package (Version 4.4.1). Raw metabolomic data were converted to mzXML format using ProteoWizard (Version 3.0.7414) and processed using an in-house program developed in R based on XCMS for peak detection, extraction, alignment, and integration. For metabolite identification, the R package (version 4.4.1) and BiotreeDB (V3.0) were used.

## 3. Results

### 3.1. Analysis of VOCs

**Figure 1:**
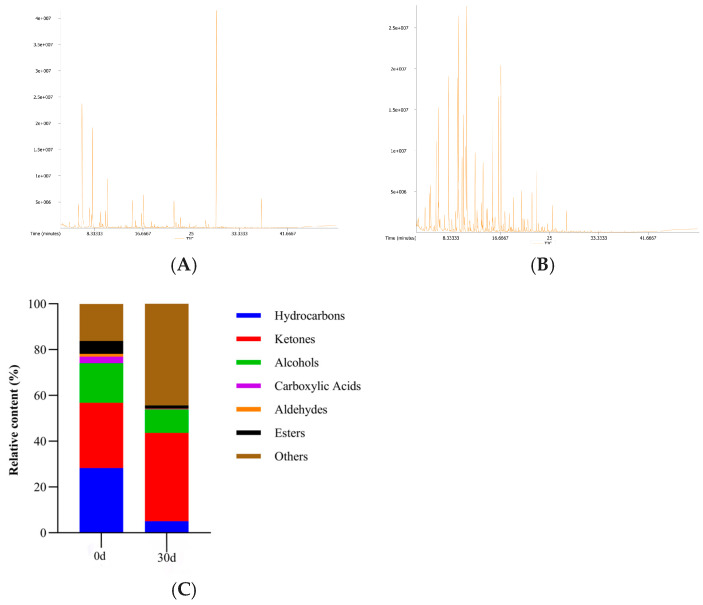
The GC-MS total ion chromatogram (TIC) of the volatile components in *Esox lucius* during superchilling ((**A**) 0 d, (**B**) 30 d) and the relative contents of volatile compounds at two time points (**C**).

The GC-MS total ion chromatogram (TIC) of the volatile components in *Esox lucius* while superchilled and the relative contents of volatile compounds at different storage times are shown in Figure 1. A total of 139 flavor-related volatile compounds were identified, including 22 hydrocarbons, 35 ketones, 43 alcohols, 16 acids, 10 aldehydes, 8 esters, and 5 other compounds. Compared to those under refrigeration, this study identified a greater number of compounds [17]. As illustrated in Figure 1C, the relative abundances of hydrocarbons, acids, alcohols, aldehydes, and esters generally declined during storage with extended micro-frozen storage durations. In contrast, ketones and other compounds, such as nitrogen- or sulfur-containing derivatives, accumulated markedly. The observed reduction in hydrocarbons is likely associated with their decreased oxidative stability under low-temperature conditions. During prolonged storage, long-chain hydrocarbons may degrade into short-chain volatiles, including ketones or aldehydes, or participate in radical-mediated lipid oxidation cascades [18]. The systemic reduction in organic acids may reflect microbial metabolic adaptations, despite transient accumulation of short-chain acids such as acetic acid during specific phases. Conversely, oxidation or esterification of medium- to long-chain fatty acids, exemplified by palmitic acid, may account for their depletion [19]. The concurrent decline in both alcohols and aldehydes suggested their dynamic interconversion serving as transient intermediates, alcohols were presumably oxidized to ketones or carboxylic acids via enzyme-mediated dehydrogenase pathways, while aldehydes may participate in Strecker degradation or react with amino compounds to form various nitrogenous heterocycles, such as pyrazines [20]. The progressive attenuation of ester levels further corroborated the temperature-dependent suppression of esterase activity, driving the ester synthesis–hydrolysis equilibrium toward net decomposition [21]. The pronounced enrichment of ketones and other compounds exhibits a dual effect on flavor attributes. Ketone accumulation, exemplified by 3-hydroxy-2-butanone typically attributed to lipid β-oxidation or Maillard reaction intermediates, imparts creamy or caramel-like aromatic notes; however, elevated levels produce undesirable sensory characteristics. Nitrogen- and sulfur-containing species within other compounds, such as pyrazines, indole, and dimethyl sulfide, demonstrate pronounced associations with spoilage-derived off-flavors, including fishy and sulfury odorants [22]. These compositional shifts implied that micro-frozen storage partially attenuated spoilage progression but inadequately suppressed the synergistic interplay between lipid oxidation and microbial metabolism, ultimately precipitating a sensory transition from fresh/mild profiles to oxidized/fermented off-odors.

Based on the screening criteria of variable importance in projection values exceeding one, a significance threshold of *p* < 0.01, and absolute logFoldChange values greater than two, 25 key flavor compounds were identified (Table 1). These included ketones, alcohols, aldehydes such as 2-methylbutanal, esters including 2-heptyl acetate and methyl butyrate, and nitrogen-containing compounds like pyrazine and indole.

Seven ketones, specifically 2-heptanone, 2-octanone, and 2-nonanone, were screened and demonstrated significant dominance within the volatile profile. This dominance was attributed to the lipid β-oxidation pathway. For instance, 2-heptanone and 2-nonanone are generated via oxidative decarboxylation of medium-chain fatty acids (C8–C12), contributing creamy or fruity notes. The formation of 3-octanone is associated with the further oxidation of Maillard reaction intermediates, such as α-dicarbonyls, particularly under conditions of high temperature or prolonged storage. Ketones not only directly influence sensory attributes but also modulate overall aroma perception through their high volatility, thereby masking the thresholds of other flavor compounds [23].

Thirteen alcohols, including representative compounds such as 1-hexanol and phenylethyl alcohol, were identified, indicating their dual functionality within the flavor system. Short-chain alcohols, exemplified by 1-pentanol and 2-pentanol, were derived from lipid peroxidation or microbial metabolism, such as that by yeast and lactic acid bacteria, and imparted grassy or floral notes to the flavor profile [22]. Long-chain alcohols, for instance, 2-pentadecanol and Z-3-dodecen-1-ol, likely originated via lipase-catalyzed hydrolysis of glycerides, with their hydrophobicity contributing to prolonged flavor persistence. Acting as intermediates in redox reactions, these alcohols were further converted primarily to aldehydes or ketones, such as the conversion of 2-nonanol to 2-nonanone, via dehydrogenase activity [24]. This transformation highlights the dynamic interplay between oxidative stress and microbial metabolism occurring during storage.

2-Methylbutanal, a critical aldehyde, was proposed to originate from Strecker degradation, such as valine deamination, or from secondary cleavage of lipid oxidation products [25]. Its low sensory threshold significantly contributed to the perception of “freshness”; however, its instability resulted in oxidation to acids or interactions with amino compounds, such as pyrazine formation, thereby accelerating flavor deterioration [26]. Concurrently, reduced levels of 2-heptyl acetate and methyl butyrate likely reflected esterase inhibition, particularly under conditions of low temperature or high water activity, which shifted the esterification-hydrolysis equilibrium toward decomposition [20]. This process weakened the fruity and floral characteristics and promoted the release of free acids, consequently exacerbating oxidative rancidity.

Pyrazine and indole highlight the role of nitrogen-containing compounds in flavor complexity. Pyrazines, formed via Maillard reactions or microbial metabolism, impart nutty/roasted aromas [27]. Indole, a microbial tryptophan metabolite, enhanced flavor “layering” at low concentrations but synergistically produced fishy/fecal off-odors with sulfides at elevated levels [28].

### 3.2. Identification and Classification of Metabolites

**Figure 2:**
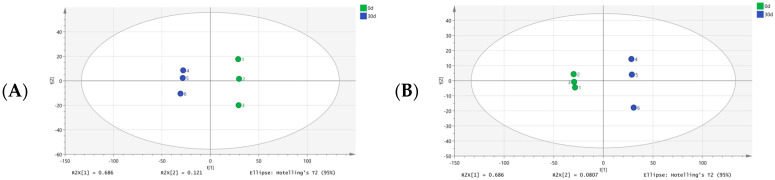

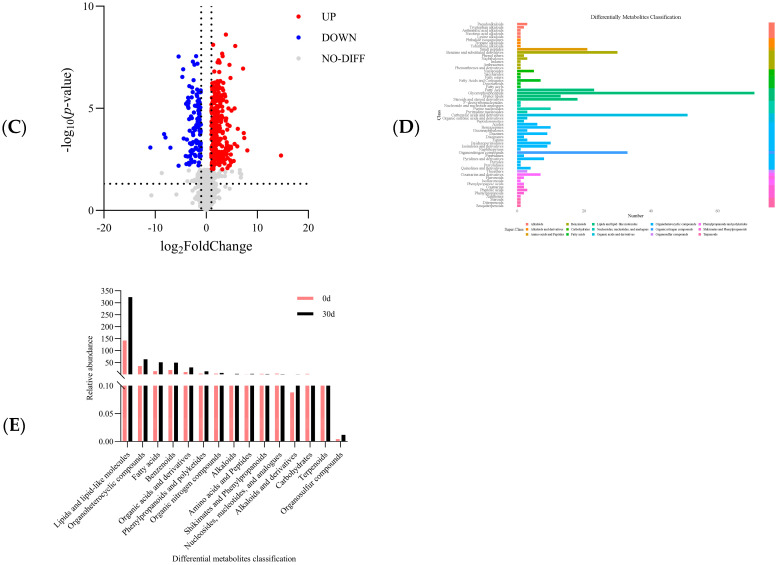
Multivariable statistics of *Esox lucius* during superchilled storage. (**A**) Principal component analysis (PCA) based on metabolomics. (**B**) PLS-DA analysis based on metabolomics. (**C**) Volcano plot. (**D**) Compound classification plot of differential metabolites. (**E**) The relative abundance of differential metabolites classification.

In total, 1174 metabolites were identified in this study using untargeted metabolomics. Principal component analysis (PCA) demonstrated that the first two principal components (PC1 and PC2) cumulatively accounted for 80.7% of the total variance (Figure 2A). A clear separation trend was observed in the score plot between 0 d and 30 d samples, indicating significant differences in metabolic profiles between the two groups. The partial least squares discrimination analysis (PLS-DA) model further validated intergroup differences, with explained variances and predictive capability confirmed by permutation testing (Figure 2B). Log_2_ fold-change (log_2_FC) values quantifyied the relative difference in compound abundance between two experimental groups, calculated as the base-2 logarithm of the ratio of mean abundances. Absolute log_2_FC values (|log_2_FC|) represent the scalar magnitude of this change irrespective of direction (positive/negative), enabling prioritization of compounds undergoing substantial concentration shifts (|log_2_FC| > 1 indicates >2-fold absolute change), while mitigating bias from symmetric data distributions common in metabolomics. Volcano plot analysis screened 466 significantly differential metabolites using thresholds of |log_2_(FC)| > 1 and *p*-value < 0.01 (Figure 2C). Among these, 347 metabolites were upregulated and 119 metabolites were downregulated in the 30 d group compared to the 0 d group. These differential metabolites were categorized into 15 major groups: alkaloids, alkaloids and derivatives, amino acids and peptides, benzenoids, carbohydrates, fatty acids, lipids and lipid-like molecules, nucleosides, nucleotides, analogs, organic acids and derivatives, organoheterocyclic compounds, organic nitrogen compounds, organosulfur compounds, phenylpropanoids and polyketides, shikimates, phenylpropanoids, and terpenoids (Figure 2D). This classification was similar to that under refrigeration conditions [29]. Among them, lipids and lipid-like molecules, organoheterocyclic compounds, and organic acids and derivatives ranked as the top three categories by proportion, accounting for 30.41, 16.78, and 13.63%, respectively.

### 3.3. Analysis of Differential Metabolites

#### 3.3.1. Differential Metabolites Related to Lipids and Lipid-like Substances

Among the identified compounds, lipids and lipid-like substances exhibited the highest relative abundance, accounting for 60.20% and 59.26% of the total content on day 0 and day 30, respectively (Figure 2E). Lipids and lipid-like substances play pivotal roles in the formation and evolution of flavor profiles during low-temperature storage of fish products. These compounds influence the overall flavor characteristics through complex biochemical reaction networks. In this study, their relative abundance was higher at day 30 compared to day 0. This phenomenon may be attributed to the enzymatic hydrolysis of triglycerides and phospholipids during the initial storage phase, catalyzed by lipases such as phospholipases and lipoprotein lipases [30]. This process releases low-molecular-weight compounds, including free fatty acids and glycerol, which subsequently undergo oxidation to generate volatile compounds such as aldehydes, ketones, and alcohols [22]. These volatiles contribute to the characteristic umami and fresh aroma. Concurrently, phosphate groups liberated from phospholipid degradation enhance umami perception. However, extended storage duration facilitated the autoxidation of polyunsaturated fatty acids, notably eicosapentaenoic acid (EPA) and docosahexaenoic acid (DHA), under low-temperature conditions [30]. This led to peroxide formation and their subsequent decomposition into short-chain aldehyde and ketone derivatives. Accumulation of these oxidative products beyond critical thresholds induced characteristic fishy-rancid off-flavors [31]. Notably, the experimentally observed biochemical alterations, particularly the dominance of lipid-derived off-flavors after 30 d of storage, unequivocally indicate sensory degradation in *Esox lucius*, marking quality deterioration.

#### 3.3.2. Differential Metabolites Related to Organoheterocyclic Compounds

Organoheterocyclic compounds primarily comprise pyrazines, furans, thiazoles, and their derivatives. Within their optimal concentration ranges, pyrazines impart desirable roasted and nutty aromas to food products, while certain sulfur-containing heterocyclic compounds can contribute seafood-like flavor characteristics [32]. However, extended storage periods lead to the gradual accumulation of oxidative derivatives from these compounds, such as dimethyl trisulfide. This accumulation can generate undesirable sulfurous off-odors and volatile compounds associated with spoilage [33]. In this study, the observed increase in relative abundance of organoheterocyclic compounds during storage may be attributed to the following mechanisms: under superchilled storage conditions, endogenous proteases in fish muscle retain residual activity, continuously hydrolyzing myofibrillar proteins to release free amino acids and reducing sugars [34]. These degradation products serve as essential precursors for both Maillard reactions and Strecker degradation, facilitating the gradual formation of heterocyclic compounds such as pyrroles and pyrazines even at subzero temperatures [35,36]. Psychrotolerant microorganisms, including *Pseudomonas* spp. present in the fish, maintain basal metabolic activity under superchilled conditions. These microorganisms secrete enzymes such as thioredoxin reductase, which catalyzes the deamination and decarboxylation of sulfur-containing amino acids, thereby promoting the biosynthesis of thiophenes and thiazoles [37,38]. Furthermore, reactive carbonyl compounds generated during lipid oxidation undergo non-enzymatic condensation reactions with amino compounds, forming more complex heterocyclic structures, for instance, furopyridines.

#### 3.3.3. Differential Metabolites Related to Fatty Acids

Fatty acids, as critical precursors for flavor formation, dynamically regulate the flavor quality of the product through oxidative degradation and enzymatic reactions [39]. Free fatty acids are continuously released under the synergistic action of lipases and psychrotolerant microorganisms with their oxidation products contributing to characteristic flavors like roasted and grassy notes while also inducing negative sensory attributes such as rancidity due to excessive accumulation of aldehydes and ketones [18,29]. The observed increase in the relative abundance of fatty acids during storage originates from the sustained activity of endogenous enzymes under low-temperature conditions, catalyzing the hydrolysis of triglycerides and membrane phospholipids to release free fatty acids and flavor precursors [40]. The acceleration of autoxidative chain reactions in polyunsaturated fatty acids (PUFAs) driven by dissolved oxygen enrichment at ice crystal interfaces, promotes the decomposition of hydroperoxides into volatile compounds [30]. In addition, the cryoconcentration effect induced by ice crystal formation, which enhances the relative enrichment of lipid components in the residual unfrozen phase as water-holding capacity declines [20]. This coupled biochemical and physicochemical process results in a higher production rate of fatty acids compared to their degradation and volatilization, ultimately leading to a significant rise in the apparent content of fatty acid components during the storage period. This variation is directly linked to the determination of flavor thresholds in fish and the endpoint of shelf life.

#### 3.3.4. Differential Metabolites Related to Benzenoids

During the low-temperature storage of fish, benzenoids regulate flavor quality through their distinctive aromatic properties. On one hand, low-threshold volatile compounds such as benzaldehyde and phenethyl alcohol impart sweet and floral characteristics to fish flesh, enhancing flavor complexity. On the other hand, the accumulation of phenolic oxidative derivatives may induce smoky off-odors or herbal bitterness, leading to the deterioration of sensory quality [41]. The observed upward trend in their relative abundance is closely associated with synergistic multifactorial mechanisms, enzymatic decarboxylation or Strecker degradation of aromatic precursors during lipid peroxidation which releases benzenoid derivatives [37]. Psychrotolerant microorganisms synthesize secondary metabolites such as phenyl acetate through the phenylpropanoid metabolic pathway under low-temperature conditions. This progression establishes benzenoids as a critical indicator of quality deterioration during superchilled storage.

#### 3.3.5. Differential Metabolites Related to Organic Acids and Derivatives

Under superchilled storage conditions, organic acids and their derivatives in the muscle tissue of *Esox lucius* shape the complexity of flavor quality through multiple pathways. Small-molecule organic acids such as lactic acid and succinic acid establish the umami foundation by modulating pH and synergizing with free amino acids, while derivatives like ethyl acetate and γ-aminobutyric acid contribute fruity aromas and umami-enhancing effects [42]. However, prolonged storage leads to the accumulation of short-chain fatty acids such as propionic and butyric acids, which may induce rancid odors [18]. The sustained increase in their relative abundance is attributed to enhanced proteolytic and lipolytic catabolism driven by the activation of endogenous proteases and lipases, promoting amino acid deamination and β-oxidation of fatty acids to generate organic acid precursors. Additionally, persistent metabolic activities of psychrotrophic microorganisms at subzero temperatures, which accelerate organic acid biosynthesis via perturbations in the tricarboxylic acid (TCA) cycle and extracellular enzymatic actions [43]. These changes not only reflect metabolic pathway imbalances within the superchilled system but also influence muscle texture through organic acid–metal ion chelation, ultimately generating characteristic “sour-fishy” composite off-flavor signals during the mid-to-late storage phases [44].

### 3.4. Screening for Key Metabolic Pathways

Based on the screening criteria of VIP > 1, a significance threshold (*p* < 0.01), and |logFoldChange| > 2, 281 key differential metabolites were identified. This study investigated metabolic regulation disparities among experimental groups through Kyoto Encyclopedia of Genes and Genomes (KEGG) pathway enrichment analysis. Based on differentially expressed metabolites screened between groups, pathway enrichment analysis was performed using the hypergeometric distribution test. Under the significance threshold (*p* < 0.01), four metabolic pathways demonstrated significant enrichment: glycerophospholipid metabolism, purine metabolism, pentose phosphate pathway, and arginine biosynthesis. These significantly enriched pathways collectively contained 27 differentially expressed metabolites (Table 2).

#### 3.4.1. Glycerophospholipid Metabolism

During micro-frozen storage, the dynamic imbalance in glycerophospholipid metabolism pathways of *Esox lucius* significantly impacts its flavor quality through multi-level molecular mechanisms. The marked accumulation of phospholipid degradation markers 1-acyl-sn-glycero-3-phosphocholine and sn-glycero-3-phosphocholine indicates enhanced phospholipase A2 (PLA2) activity, triggering deacylation of membrane phospholipids [45]. This process induces structural disintegration of the phospholipid bilayer, releasing free fatty acids into the lipoxygenase pathway that catalyzes the formation of short-chain carbonyl compounds such as hexanal and pentanal, imparting characteristic oxidative rancidity to fish muscle [46]. Concurrently, elevated levels of the phospholipid degradation intermediate phosphocholine suggest potential conversion through choline oxidation pathways to trimethylamine oxide (TMAO), which undergoes microbial reduction during prolonged storage to generate trimethylamine (TMA) with intense fishy odor [47]. The abnormal accumulation of CDP-ethanolamine indicates obstruction in phosphatidylethanolamine biosynthesis, compromising membrane repair mechanisms. This deficiency accelerates ATPase leakage and induces calcium-dependent degradation of myofibrillar proteins, generating bitter-tasting peptides. Furthermore, the significant depletion of phosphatidylcholine—a critical membrane structural component—and its metabolic intermediate sn-glycero-3-phosphoethanolamine directly increases myocyte membrane permeability [48]. This structural alteration promotes leakage of umami-related nucleotides and accelerates hemoglobin release. Subsequent oxidation of hemoglobin under low-temperature storage conditions generates verdoglobin, contributing to characteristic green-brown discoloration [49]. These cascading events ultimately synergize through lipid oxidation products, protein degradation derivatives, and microbial metabolites, collectively forming undesirable flavor profiles dominated by rancid and putrid odors [50]. The integrated effects of membrane phospholipid metabolism dysregulation, oxidative stress responses, and microbial activity comprehensively explain the deterioration mechanisms of sensory attributes in cryopreserved *Esox lucius* muscle.

#### 3.4.2. Purine Metabolism

The upregulation of ADP in the purine metabolic pathway reflects the continuous degradation of ATP catalyzed by adenylate kinase (AK), suggesting a disturbance in the energy metabolism system [51]. This metabolic dysregulation may lead to decreased structural stability of myofibrillar proteins, potentially associated with reduced fish body firmness and textural deterioration during later storage stages. The elevated levels of hypoxanthine and xanthine, which are catalyzed by xanthine oxidase (XOD) to generate uric acid, not only produce bitter-tasting substances but also promote reactive oxygen species (ROS) accumulation [52]. This oxidative stress subsequently triggers lipid oxidation, yielding aldehyde and ketone compounds responsible for off-flavor development. Concurrently, the downregulation of key umami-enhancing nucleotides, inosine monophosphate (IMP) and guanosine monophosphate (GMP), directly diminishes umami intensity. The reduction in adenosine and guanine further compromises the synergistic flavor profile by weakening the complementary sweet and umami taste dimensions [53]. This metabolic reprogramming may be closely associated with enzymatic system imbalances during storage, characterized by increased acid phosphatase (ACP) activity and suppressed 5′-nucleotidase (5′-NT) functionality [54,55]. Such biochemical alterations ultimately contribute to umami loss, bitterness intensification, and the generation of undesirable odor compounds.

#### 3.4.3. Pentose Phosphate Pathway

In the pentose phosphate pathway (PPP), the levels of D-ribose, D-glycerate, D-fructose 6-phosphate, and D-gluconic acid were upregulated. The accumulation of D-ribose, as a Maillard reaction precursor, may facilitate the formation of characteristic flavor compounds during later storage stages. However, excessive accumulation could accelerate protein glycosylation, potentially leading to reduced muscle elasticity [56]. The enrichment of D-fructose 6-phosphate suggests a metabolic shift toward this pathway within the carbohydrate network, which might reduce the availability of key glycolytic intermediates, thereby slowing lactate accumulation and mitigating pH decline-induced deterioration of muscle water-holding capacity [57]. The concurrent elevation of D-gluconic acid and D-glycerate indicates dual metabolic implications, activation of the NADPH/GSH antioxidant system, which helps suppress lipid peroxidation-derived off-flavors, and potential metabolic reprogramming in organic acid metabolism that may influence myofibrillar protein stability through altered cellular osmotic pressure [58]. This metabolic remodeling demonstrates a balance between maintaining fundamental flavor stability in fish muscle and generating characteristic accumulations of mildly sweet precursors. The observed metabolic adjustments suggest a coordinated response to storage stress, where flavor preservation mechanisms coexist with subtle textural modifications mediated through protein–organic acid interactions.

#### 3.4.4. Arginine Biosynthesis

During the micro-frozen storage of *Esox lucius*, significant metabolic perturbations were observed in the arginine biosynthesis pathway, manifested by marked upregulation of glutamate and citrulline accompanied by synchronous downregulation of fumaric acid. Glutamate accumulation, characteristic of this umami amino acid, initially enhanced the umami profile of fish flesh during the early stages of storage. However, excessive accumulation subsequently underwent deamination, forming volatile nitrogen-containing compounds such as ammonia and trimethylamine. This process potentially contributed to off-flavor intensification in later storage phases. Elevated citrulline levels indicated activation of arginine synthesis pathways. The redirection of metabolic flux promoted the formation of protein oxidation products via the arginine–proline metabolic axis, thereby accelerating textural deterioration of muscle tissue [36]. Notably, the downregulation of fumaric acid, a crucial tricarboxylic acid (TCA) cycle intermediate, not only induced an energy metabolism imbalance but also disrupted glutamate homeostasis by reducing α-ketoglutarate availability. This metabolic disturbance could exacerbate the accumulation of lipid peroxidation end products, including hexanal and nonanal, ultimately generating the characteristic frozen odor [59]. The synergistic effects of these metabolic reprogramming events, namely umami substance depletion, off-flavor compound formation, and texture softening, collectively drove the deterioration of overall flavor quality in frozen-stored pike specimens.

## 4. Conclusions

This study demonstrated that superchilled storage significantly modulates the flavor quality evolution of *Esox lucius* through the regulation of key metabolic networks. Integrated analysis combining GC-MS and non-targeted metabolomics revealed that the dynamic changes of 25 characteristic volatile compounds, such as aldehydes, ketones, and nitrogen-containing compounds, exhibited strong correlations with four critical metabolic pathways: glycerophospholipid metabolism, which regulates lipid oxidation; purine metabolism, which mediates nucleotide degradation; the pentose phosphate pathway, associated with attenuated antioxidant capacity; and arginine biosynthesis, indicative of amino acid metabolic imbalance. Notably, the synergistic interaction between glutamate accumulation and fumarate depletion within the arginine metabolic pathway drove the formation of a characteristic undesirable frozen odor, while lipid oxidation derivatives showed a direct correlation with muscle texture deterioration. From a metabolic perspective, this study elucidates the molecular mechanisms underlying flavor quality deterioration during superchilled storage, providing a theoretical foundation for targeted regulation to optimize preservation techniques for cold-water fish species.

This study has limitations. First, the relatively small sample size (n = 16) constrained by collection conditions may affect the generalizability of metabolomic findings. Second, the absence of systematic sensory evaluation within the research framework limits the interpretation of associations between chemical profiles and flavor perception. Future work will expand sample cohorts and integrate multimodal approaches (human sensory panels, electronic tongue/nose) to establish robust chemosensory correlation models, with cross-regional sampling to enhance extrapolation validity. In addition, while this work establishes time-dependent flavor profiles within 30 days of storage, the stability of key biomarkers (TAV-active aldehydes, nucleotides) under ultra-long-term superchilling warrants targeted investigation to optimize logistics for distant markets.

## Figures and Tables

**Table 1:** Volatile compounds relative content of *Esox lucius* detected by GC-MS during superchilled storage.

Volatile Compounds	RT (min)	Relative Abundance (%)		VIP	*p*-Value	Log_2_Foldchange
		0 d	30 d			
1-Hexanol	14.59	1.83	ND	1.25	0.001	−12.513
(Z)-3-Dodecen-1-ol	14.86	1.14	ND	1.251	0.006	−11.831
1-Pentanol	11.73	0.78	ND	1.25	0.003	−11.284
2-Methyl-butanal	2.94	0.52	ND	1.255	0.010	−10.682
Phenylethyl Alcohol	27.53	29.12	0.16	1.22	0.001	−4.0467
6-Methyl-5-hepten-2-ol	17.46	0.16	0.13	1.22	0.000	3.2401
2-Heptanone	9.44	41.30	38.08	1.16	0.0011	3.366
2-Nonanone	15.39	18.32	21.58	1.21	0.001	3.719
3-Octanone	11.55	0.38	0.52	1.25	0.001	3.946
2,2-Dimethyl-3-octanone	29.61	0.05	0.11	1.05	0.001	4.633
(E)-6-Nonen-1-ol	19.76	0.09	0.23	1.00	0.000	4.810
2-Octanone	12.46	4.75	13.69	1.23	0.000	5.011
3-Heptanone	8.52	0.01	0.03	1.11	0.000	5.436
2-Nonanol	18.96	1.33	5.98	1.22	0.000	5.649
6-Methyl-2-heptanol	15.22	0.17	0.77	1.003	0.0030	5.7040
2-Decanol	20.31	ND	0.04	1.25	0.002	9.676
2-Pentadecanol	25.07	ND	0.07	1.25	0.009	10.726
2-Heptyl acetate	11.90	ND	0.10	1.25	0.001	11.236
3-Nonanol	18.29	ND	0.11	1.25	0.004	11.299
Indole	37.13	0.05	15.86	1.24	0.003	11.920
3-Octanol	15.73	ND	0.29	1.25	0.000	12.723
Methyl butyrate	4.15	ND	0.41	1.251	0.009	13.222
2-Pentanol	8.14	ND	0.48	1.25	0.007	13.446
Pyrazine	10.30	ND	0.55	1.25	0.002	13.645
2-Hexanone	6.48	ND	0.80	1.25	0.005	14.178

Note: Variable importance in projection (VIP) ≥ 1 indicated that this compound was an important flavor compound. ND, not detected.

**Table 2:** Differently expressed metabolites in significantly enriched pathways.

ID	Name	RT	Relative Abundance		VIP	*p*-Value	Log_Foldchange	Up/Down
			0 d	30 d				
1	IMP	256.5	2.075410253	0.172349174	1.305	0.000	−3.589	down
2	GMP	263.7	0.157221115	0.014306665	1.308	0.000	−3.458	down
3	Imidazoleacetic acid	191.3	0.402646941	0.054648313	1.34	0.004	−2.881	down
4	Adenosine	85.4	0.505515164	0.081046236	1.34	0.000	−2.640	down
5	Deoxyadenosine	58.9	0.019532804	0.003480467	1.33	0.000	−2.488	down
6	Carnosine	246.5	1.248932617	0.34588395	1.34	0.000	−1.852	down
7	Guanine	139.8	4.12418659	1.466873231	1.34	0.000	−1.491	down
8	Fumaric acid	215.5	0.043522088	0.016290857	1.35	0.000	−1.417	down
9	Dimethylglycine	181.3	0.005597956	0.011406206	1.34	0.000	1.026	up
10	Hypoxanthine	82.5	15.10863255	31.39561676	1.34	0.000	1.055	up
11	ADP	264.5	0.014330009	0.030932311	1.34	0.000	1.110	up
12	dGDP	264.5	0.014330009	0.030932311	1.34	0.000	1.110	up
13	Deoxyinosine	95.9	0.182593159	0.410807127	1.355	0.000	1.169	up
14	CDP-Ethanolamine	83.8	0.207212681	0.492886833	1.34	0.003	1.250	up
15	Xanthosine	184.2	0.007277305	0.017887743	1.24	0.002	1.297	up
16	Xanthine	123.5	0.631407431	1.773516428	1.34	0.000	1.489	up
17	1-Acyl-sn-glycero-3-phosphocholine	19.5	2.108224746	6.131611592	1.35	0.000	1.540	up
18	Glutamate	229.1	0.086630744	0.294221196	1.32	0.000	1.763	up
19	Uric acid	191.2	0.006596723	0.023092878	1.33	0.000	1.807	up
20	Pyruvate	47.5	3.782425277	13.2657556	1.34	0.000	1.810	up
21	Gluconic acid	227.4	0.084084384	0.545685796	1.31	0.000	2.698	up
22	Glyceric acid	179	0.01225653	0.082420234	1.32	0.000	2.749	up

## Data Availability

The original contributions presented in the study are included in the article, further inquiries can be directed to the corresponding author.
